# Gravity and neuronal adaptation, in vitro and in vivo—from neuronal cells up to neuromuscular responses: a first model

**DOI:** 10.1007/s00249-017-1233-7

**Published:** 2017-06-27

**Authors:** Florian P. M. Kohn, Ramona Ritzmann

**Affiliations:** 1 Department of Membrane Physiology (230b), Institute of Physiology (230), University of Hohenheim, Garbenstr. 30, 70599 Stuttgart, Germany; 2 Institute of Sport and Sport Science, University of Freiburg, Freiburg, Germany

**Keywords:** Microgravity, Hypergravity, Neuronal system, Adaptation, Sensorimotor function, Membrane properties, Electrophysiology

## Abstract

For decades it has been shown that acute changes in gravity have an effect on neuronal systems of human and animals on different levels, from the molecular level to the whole nervous system. The functional properties and gravity-dependent adaptations of these system levels have been investigated with no or barely any interconnection. This review summarizes the gravity-dependent adaptation processes in human and animal organisms from the in vitro cellular level with its biophysical properties to the in vivo motor responses and underlying sensorimotor functions of human subjects. Subsequently, a first model for short-term adaptation of neuronal transmission is presented and discussed for the first time, which integrates the responses of the different levels of organization to changes in gravity.

## Introduction

Of the four fundamental interactions (strong interaction, weak interaction, electromagnetic force, and gravity), gravity is the weakest. Nevertheless, gravity is responsible for the formation of stars and planets as the sun or earth (Montmerle et al. 2006). During the development of life, many properties on earth changed such as solar irradiation, temperature, humidity, etc., but the gravity field remained constant since the final stages of planet formation. Therefore, since millions of years, earth life developed and adapted to these persistent 1-g conditions.

This permanent gravity stimulus led to various gravity-perceiving systems in organisms that influence, i.e., movement and behavior or growth on earth. Gravity dependencies have been described on the molecular, cellular, and complex structural level of organisms: thereby, one of the most intensively researched systems in humans and animals is the nervous system (NS) that is—beside others—crucial for movement control, sensory integration, and terrestrial locomotion of earth species. The NS governs muscle contraction enabling the body to counteract the gravitational force as a physical impact and controlling typical body motion and locomotor patterns as during the evolutionary shift from aqueous to terrestrial life. The NS consists of interconnected neurons and supporting glial cells. Neuronal communication is based on electrochemical coupling, the modulation of intra- and extracellular ions to modify the electrical properties of a cell (intracellular signaling), and the controlled release of transmitters (intercellular communication). On the complex level, one of the most fundamental circuitries within the CNS is the reflex arch (Ritzmann et al. 2016). Spinal reflexes are simple neuromuscular reactions in response to a stimulus providing fast muscle contractions occurring with a delayed magnitude proportional to the sensory input integrated into movement. Allowing mobility of terrestrial life, sensory input from the vestibular and visual systems and proprioception is processed by the NS and by means of muscle innervation, appropriate forces are generated to control simple posture or movement (Margaria and Cavagna 1964; Layne et al. 2001; Ritzmann et al. 2015; Bloomberg et al. 1999; Homick and Reschke 1977; Paloski et al. 1993). Life is based on these sensorimotor competencies.

Decades of space research made gravity-induced changes in the NS apparent, and since the first manned space missions, the effect of microgravity on humans has been investigated, as various effects on astronauts and cosmonauts have been observed. With an emphasis on weightlessness and our astronomical neighbors Mars and the moon (Margaria and Cavagna 1964; Spudis 1992), authors found directly related health effects, among others a persistent modulation in the sensory (Paloski et al. 1993; Reschke et al. 1986) and motor system (Blottner and Salanova 2015) and the resulting structural loss of muscle (Di Prampero and Narici 2003) and bone mass (Loomer 2001). In addition, there are modulations in the neuromuscular system underlying those health-related changes that open up a lot of questions on how gravity, and the absence of it, influences the NS. These questions led to numerous experiments to investigate the effect of varying gravity conditions on the different levels of organization, from the molecular and cellular level up to the whole NS and the interconnection with movement control and mobility. The functional properties of these levels were thoroughly investigated, however, with barely any interconnection.

The aim of this review is to give an overview of the acute gravity-dependent adaptations of the NS of humans and animals from the molecular level up to the sensorimotor systems and to present and discuss a first model of neuronal short-term adaptation that takes into account the findings on the different levels of organization. This has been done on the basis of in vitro and in vivo studies executed in varying gravity environments. Consequences and prospects for space missions and countermeasure applications were integrated. Regarding the gravity-dependency on a functional level of human organisms, beside direct motor responses of single nerves, the monosynaptic reflex arc (Crone et al. 1990; Zehr 2002) have been selected for a functional description with focuses on afferent and efferent pathways. Even though many ongoing experiments are focusing on the human brain (e.g., the NEUROMapping program from NASA), the brain and its sub-compartments have been excluded from the analysis as the interpretation of the different studies is quite challenging and should be addressed separately.

## Methods

### Literature search

We performed a computerized systematic literature search in PubMed and Web of Knowledge from January 1950 up to February 2016. Keywords were included in our final Boolean search strategy as follows: ‘space’ OR ‘parabolic flight’ OR ‘rocket’ OR ‘drop tower’ AND ‘neuro’ PR ‘neuron’ OR ‘ion channel’ OR “action potential” OR ‘sensorimotor’ OR ‘reflex’ OR ‘latency’ OR ‘neuromuscular’. The search was limited to English and German languages, cells and human species, and to full-text original articles, books, and conference abstracts. We scanned each article’s reference list in an effort to identify additional suitable studies for inclusion in the database.

### Selection criteria

To be eligible for inclusion, studies had to meet the following criteria: experiments had to be executed in real microgravity conditions in either space missions (MIR, ISS, or Shuttle), parabolic flights, sounding rockets, or drop tower. Studies were excluded if experiments were performed in simulation studies (random positioning machine, clinostat, bed rest, immobilization, water immersion, and partial weight bearing) under the influence of gravitational acceleration due to confounding side effects.

Inclusion criteria for cell biology were as follows: (1) clear experiment design (2), related to (3) neuronal and (4) neuromuscular effects, and (5) related molecular analyses.

Human life science studies had to meet the following criteria: (1) controlled study design related to (2) neuromuscular effects executed in (3) participants had to be healthy with an age range of 18–70 years.

### Coding of studies

Each study was coded for the following variables for cell physiology: setting (parabolic flight, sounding rocket, space flight) gravity conditions (hypo, normal, hyper), neuronal properties (action potential, resting potential), ion channels (open state, closed state, conductivity), biophysical properties, membrane properties.

The following variables have been selected for human life science studies: type of study (cross-sectional, longitudinal), setting (parabolic flight, space flight), gravity conditions (hypo, normal hyper), nerve (sensory, motor, sensory motor interconnection).

## Results

### In vitro experiments

Due to the complexity of the experiments, most of the experiments have been performed on short-term gravity research platforms like the parabolic flight missions or drop towers.

A summarizing table of the used literature is given at the end of the in vitro chapter (Table 1).

**Table 1:** A short summary of the literature: the used gravity conditions, methodology, and the outcome

Authors (year)	Gravity conditions			Methodology			Outcome
	Micro-gravity	Normal	Hyper-gravity	Target	Parameter	Method	
Goldermann and Hanke (2001)	0 g	1 g	Up to 2.2 g	Porin channel (from *Escherichia coli)*	Open state probability	Artificial planar bilayer	0 g < 1 g < 2.2 g
Kohn (2012)	0 g	1 g	1.8 g	SH-SY5Y cells (human, neuronal)	Membrane potential	Fluorescence intensity	0 g > 1 g > 1.8 g
Meissner and Hanke (2005)	0 g	1 g	1.8 g	N. ischiadicus (*Rattus norvegicus*)	Propagation velocity	Electro-physiology	0 g < 1 g < 1.8 g
				Earthworm (*Lumbricus terrestris*)	Propagation velocity	Electro-physiology	0 g < 1 g < 1.8 g
	0 g	1 g	n.a.	Retzius neurons (*Hirudo medicinalis*)	Rate of action potentials	Patch-clamp	0 g > 1 g
Schaffhauser et al. (2011)	0 g	1 g	1.8 g	Oocytes (*Xenopus laevis)*	Transmembrane current	Electro-physiology	0 g < 1 g < 1.8 g
Sieber et al. (2014)	0 g	1 g	1.8 g	Asolectin vesicles SH-SY5Y cells	Membrane viscosity Membrane viscosity	Polarization anisotropy Polarization anisotropy	0 g < 1 g < 1.8 g 0 g < 1 g < 1.8 g
Wiedemann et al. (2003)	0 g	1 g	Up to 6 g	Alamethicin (from *Trichoderma viride*)	Pore frequency	Artificial planar bilayer	0 g < 1 g < 6 g
Wiedemann et al. (2011)	0 g	1 g	1.8 g	SF-21 cells (insect)	Membrane potential	Fluorescence intensity	0 g > 1 g > 1.8 g

## Subcellular parameters

### Ion channel parameters

Up to now, all experiments investigating ion channel parameters like open and closed state probability have been performed with ion channels or pore forming peptides that were reconstituted into artificial planar lipid bilayers.

It was shown that a porin channel from *Escherichia coli* has a clear gravity dependence (Goldermann and Hanke 2001). Under microgravity conditions, the mean open state is significantly decreased; at increased gravity conditions, the mean open state is increased. This effect is also fully reversible. The conductance of this porin channel was not affected significantly.

A second model system used is alamethicin, a pore-forming peptide from *Trichoderma viride*. Similar to the *E. coli* porins, the activity of alamethicin is increased towards higher gravity (>1 g) and is decreased towards microgravity (Klinke et al. 2000; Wiedemann et al. 2003).

### Membrane parameters

Biological cell membranes are complex structures and are mainly composed of lipids and proteins (Pollard and Earnshaw 2008). In neurons, the functional changes to modify the membrane potential are usually attributed to the integrated membrane proteins, the ion channels and ion pumps. Nevertheless, it is well established that parameters of the lipid matrix are directly modifying the function of proteins (Lee 2004). For the sensorimotor system, i.e., it has been shown that the closed state probability of nicotinic acetylcholine receptor channels increased towards an amplified membrane viscosity (Zanello et al. 1996).

As single neuronal cells do not have a specific gravity-sensing structure, a logical experiment is to monitor membrane properties under conditions of variable gravity. Experiments with an adapted 96-well plate reader have been performed and it was shown that membrane viscosity clearly shows a gravity dependence. Under microgravity conditions, membrane viscosity is significantly decreased (the membrane is getting more fluid), under conditions of 1.8 g, the viscosity is significantly increased (the fluidity is decreased). Membrane viscosity of artificial asolectin vesicles and of human SH-SY5Y cells have been investigated and both samples show a similar gravity dependence, but in a different distinctness (Sieber et al. 2014). It is assumed that the cytoskeleton or lipid composition might explain the difference in the gravity-dependent changes of membrane viscosity, but this has to be verified in future experiments.

This finding potentially has a huge impact on cellular experiments, as this effect might be a basic mechanism of how single cells detect changes in gravity, without having dedicated sensory structures.

### Cellular parameters

The electrophysiological properties of various cell types have been investigated with different methods.

It was shown that the resting potential of human neuronal cells is slightly depolarized by 3 mV under microgravity and slightly hyperpolarized under hypergravity conditions (Kohn 2012).

A similar depolarization under microgravity was observed in SF-21 cells (Wiedemann et al. 2011). Electrophysiological experiments with oocytes from *Xenopus laevis* also show a significant decrease in transmembrane current at a holding potential of −100 mV during microgravity and show a trend of increased transmembrane currents at hypergravity (Schaffhauser et al. 2011).

The changes in electrophysiological properties are very fast and reversible, they change within milliseconds as soon as the gravity is changed and return to normal when gravity returns to 1 g.

### Action potentials

Two parameters of action potentials (AP) were analyzed. In spontaneous spiking leech neurons, it was shown that the rate of action potentials is increased under microgravity (Meissner and Hanke 2005).

To monitor the propagation velocity of action potentials, intact earthworms, isolated earthworm, and rat axons have been used. All three systems show a similar, with varying degree of significance, decrease in AP velocity under microgravity and an increase in AP velocity at hypergravity. Similar to the cellular and subcellular level, the changes are very fast and reversible.

## Summary 1: in vitro experiments

### In vivo experiments

Based on the knowledge that molecular and cellular properties in neurons are modulated by gravity, complex life science studies were conducted to describe gravity-induced neuroplasticity in humans using micro- and hypergravity research platforms in parabolic flight campaigns or during long-term space missions with a duration of 10 days up to 1.5 years in human subjects. Stimulation techniques such as peripheral nerve stimulation (PNS) have been applied in order to gather a deeper understanding of microgravity-induced deconditioning in motor control (Crone et al. 1990; Zehr 2002). In those methodological approaches neurons, axons or cell bodies are depolarized and muscle membrane potentials serve for the interpretation of output signals. The nerve *tibialis posterior* and muscle *soleus* have been used in terms of a model to describe overall adaptation to micro-, hypo-, or hypergravity in most experiments. Changes in characteristics of neuromuscular responses, displayed as H-reflexes have been described according to their attributes related to timing and shaping (Ritzmann et al. 2016): stimulation threshold, amplitude neuromuscular latency, and inter peak interval. A summarizing table of the used literature is given at the end of this chapter (Table 2).

**Table 2:** A short summary of the literature: the used gravity conditions, methodology, and the outcome

Authors (year)	Gravity conditions			Human subjects		Methodology			Outcomes	
	Micro-gravity	Normal	Hyper-gravity	*N*	Age (years)	Target (N. tibialis)	Muscle	Method	Parameter	Result
Kramer et al. (2013)	0 g	1 g	1.8 g	n.a.	n.a.	Ia afferent	M. Soleus, M. Gastrocnemius medialis	PNS	H/M ratios	0 g = 1 g = 1.8 g
Miyoshi et al. (2003)	0 g	1 g	1.8 g	3	24–38	Ia afferent	M. Soleus	PNS	H-Reflex amplitude	0 g > 1 g < 1.8 g
						Efferent	M. Soleus	PNS	M-wave amplitude	0 g = 1 g = 1.8 g
Nomura et al. (2001)	0 g	1 g	1.5 g	4	22–52	Ia afferent	M. Soleus	PNS	Amplitude	0 g > 1 g = 1.5 g = 2 g
						Efferent	M. Soleus	PNS	Amplitude	0 g = 1 g = 1.5 g = 2 g
Ohira et al. (2002)	0 g	1 g	1.5 g, 2 g	n.a.	n.a.	Ia afferent	M. Soleus	PNS	Amplitude	0 g > 1 g = 1.8 g = 2.0 g
							M. Soleus	PNS	Latency	0 g = 1 g = 1.8 g = 2.0 g
						Efferent	M. Soleus	PNS	Amplitude	0 g = 1 g = 1.8 g = 2.0 g
							M. Soleus	PNS	Latency	0 g = 1 g = 1.5 g = 2.0 g
Ritzmann et al. (2015)	0 g, 0.16 g, 0.38 g	1 g	1.8 g	10	31 ± 4	Ia afferent	M. Soleus	PNS	H-Reflex amplitude	0.16 g < 0.38 g < 1 g < 1.8 g
						Efferent	M. Soleus	PNS	M-wave amplitude	0.16 g = 0.38 g = 1 g = 1.8 g
Ritzmann et al. (2016)	0.16 g, 0.38 g	1 g	1.8 g	10	24.38 years	Ia afferent	M. Soleus	PNS	Threshold	0.16 g > 0.38 g > 1 g > 1.8 g
						Efferent	M. Soleus	PNS	Threshold	0.16 g > 0.38 g > 1 g > 1.8 g
						Ia afferent	M. Soleus	PNS	Latency	0.16 g > 0.38 g > 1 g > 1.8 g
						Efferent nerve	M. Soleus	PNS	Inter-peak-interval	0.16 g > 0.38 g > 1 g > 1.8 g
						Ia afferent	M. Soleus	PNS	Inter-peak-interval	0.16 g > 0.38 g > 1 g > 1.8 g
						Efferent	M. Soleus	PNS	Amplitude	0.16 g < 0.38 g < 1 g < 1.8 g
Sato et al. (2001)	0 g	1 g		n.a.	n.a.	Ia afferent	M. Soleus	PNS	H/M ratios	0 g > 1 g

### Threshold

Changes in threshold levels to depolarize an axon or nervous cell body describes the responsiveness of a nerve to the input stimulus. Threshold data exist for short-term micro- and hypergravity. Higher stimulation currents were necessary for PNS to depolarize axons of efferent and afferent neurons in gravity conditions equal to the moon and Mars corresponding to 0.16 and 0.38 g, respectively. In hypergravity, smaller stimulation currents were necessary to depolarize the axons (Ritzmann et al. 2016). Thus, in microgravity the threshold is increased; in hypergravity the threshold is decreased.

### Amplitude

The amplitude describes the output signal after peripheral nerve stimulation. Gravity dependency has been reported in cross-sectional study designs with neuroplastic changes for amplitudes of H-reflexes and stretch reflexes (Ritzmann et al. 2015; Sato et al. 2001; Miyoshi et al. 2003; Nomura et al. 2001; Ohira et al. 2002; Kramer et al. 2013). Independently of stimulation methodology, the peak-to-peak amplitudes and integrals increased when acutely exposed to hypergravity in parabolic flight maneuvers (Ritzmann et al. 2015; Miyoshi et al. 2003).

For reduced-gravity conditions, study results are equivocal: lunar and Martian gravity studies revealed a gradual decrease in peak-to-peak amplitudes of *H*_max_ with decreasing gravitation (Ritzmann et al. 2016). However, microgravity caused either an increase in H-reflex amplitude (Sato et al. 2001; Miyoshi et al. 2003; Nomura et al. 2001; Ohira et al. 2002) or revealed no changes (Ritzmann et al. 2015; Kramer et al. 2013). An ISS experiment executed by Watt (2003) in weightlessness documented a decline of H-reflexes in space. These adaptations persisted during 5 months of weightlessness upon returning to earth and recovered the days after.

Threshold adaptations most probably caused the inhomogeneous findings observable in H-reflex amplitudes due to differences in methodology (Ritzmann et al. 2016). As M-wave and H-reflex amplitudes depend on the stimulation threshold, the increase in H-reflex amplitudes should be interpreted on the basis of threshold declines in microgravity when H-reflexes are recorded with a constant stimulation intensity (Sato et al. 2001; Miyoshi et al. 2003; Nomura et al. 2001; Ohira et al. 2002). While H/M recruitment curves are independent of the stimulation threshold (Ritzmann et al. 2015; Kramer et al. 2013), gravity-induced changes in H-reflexes elicited submaximally with a constant stimulation threshold result rather from threshold shifts than gravity changes (Sato et al. 2001; Miyoshi et al. 2003; Nomura et al. 2001; Ohira et al. 2002).

### Neuromuscular latency

Neuromuscular latency describes the axonal and/or nerve conduction velocity until a muscle response is observable in the electromyogram. Various experiments investigated the latency of the H-reflex and M-wave in the Soleus muscle in settings of short- (Ohira et al. 2002; Ritzmann et al. 2016) and long-term (Ruegg et al. 2003) varying gravity with equivocal findings: with gradually decreasing gravity from hyper- to earth to Martian to lunar gravity, Ritzmann et al. demonstrated in eight subjects an increase in latencies of H-reflexes while M-wave latencies likewise showed a strong tendency to increase towards microgravity (Ritzmann et al. 2016). In contrast, exposure to micro- or hypergravity showed no short-term effects in H-reflex and M-wave latencies in experiments executed by Ohira et al. (Ohira et al. 2002). The authors did not state the sample size.

### Inter-peak-interval (IPI)

The IPI between the negative and positive maxima of the biphasic amplitude describes the conduction velocity along the muscle fibers via the motor endplates at the neuromuscular junction where the nerve interconnects with the muscle. Short-term experiments executed in parabolic flights revealed that IPIs significantly increase for the biphasic m. Soleus *M*_max_ and *H*_max_ with decreasing gravitation from hyper- to earth to Martian to lunar gravity conditions (Ritzmann et al. 2016).

## Summary 2: in vivo experiments

### A first model of neuronal short-term adaptation to microgravity

The proposed model aims to integrate the results from the cellular level up to the neuromuscular interface. To exclude possible adaptation processes, it only contains data from short-term experiments.

### Molecular level

**Fig. 1:**
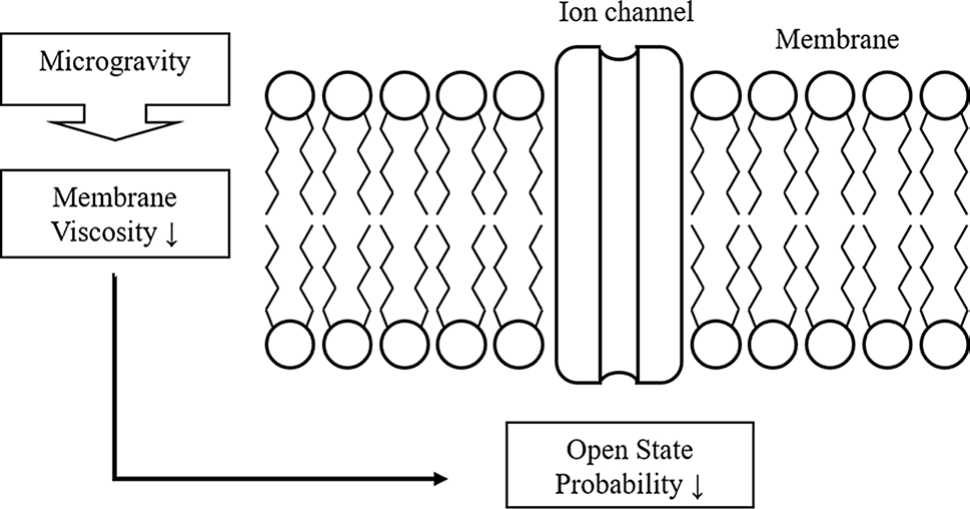
A model of the biophysical gravity dependence of cell membranes and the incorporated ion channels. With the onset of microgravity, the membrane viscosity is decreased and the open-state probability of ion channels is decreased

On the molecular level, gravity has an effect on both the membrane and integrated functional membrane proteins including ion channels. Under microgravity conditions, the membrane viscosity is decreased (the fluidity is increased). This changed membrane viscosity decreases the open-state probability of ion channels (Fig. 1). At hypergravity, these effects are inversed: membrane viscosity increases and the open-state probability increases.

Non-space related biophysical experiments clearly show that ion channel properties are dependent on membrane parameters such as lateral pressure. For alamethicin, it is known that the open state of the pore clearly depends on the lateral pressure of the membrane (Hanke and Schluhe 1993), with increased pressure, the activity increases. For other ion channels it was also shown that ion channel parameters are affected by changes in lateral membrane pressure, e.g., the closed-state probability of nicotinic acetylcholine receptor channels increases towards increased membrane viscosity (Zanello et al. 1996).

### Resting potential

**Fig. 2:**
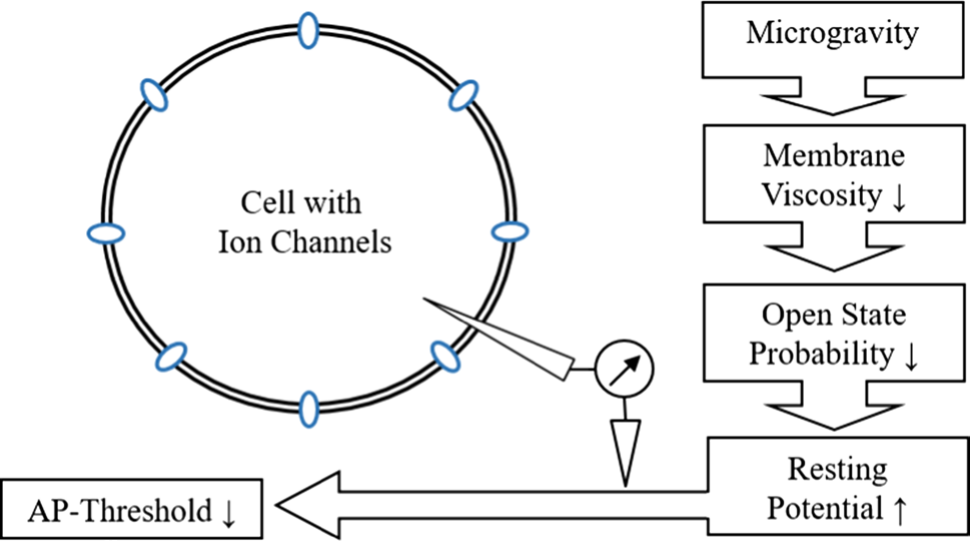
The extended model of the cellular gravity dependence of a single neuronal cell. Due to the changed membrane viscosity and the changed open-state probability, the cell depolarizes several mV. This leads to a decreased potential difference between the resting potential and the AP threshold, therefore action potentials can be triggered more easily

The resting potential of single cells is depolarized several millivolts under microgravity and hyperpolarized under hypergravity. With a slightly increased resting potential, the threshold to trigger an action potential (AP) can be reached more easily (Fig. 2). In spontaneously spiking neurons, this gravity-dependent effect was found. The rate of APs is increased in microgravity.

### Propagation of action potentials

In isolated single axons as well as in living animals and in human test subjects, the effect of microgravity can clearly be seen, the propagation speed of APs decreases under microgravity and increases under hypergravity.

In humans, the properties of neuromuscular reflexes are affected by microgravity. The latencies are increased, which can be interpreted as a decreased conduction speed. The peak-to-peak amplitude of the H-reflex is decreased under reduced gravity (with heterogenous data at real microgravity) and a higher stimulus has to be given to get the same *H*_max_ as in 1 g. The stimulation and recording method cannot be compared directly to single cell patch-clamp experiments, but the effect might be explained by a decreased propagation velocity along the axon in microgravity compared to 1-g conditions: less action potentials per time stimulate muscle contraction and therefore *H*_max_ is decreased. This interpretation is supported by the decrease in IPI under microgravity, which indicates a decreased signal speed at the neuromuscular junction. All these findings are also reversed under hypergravity.

The previously described effects can be summarized as a gravity-dependent decrease in neuronal conduction velocity (or as an increase in electrical and chemical time constants) under reduced gravity with an increase under hypergravity.

At first glance, it might look like an inconsistency that at the same time the rate of action potentials is increased in microgravity but the propagation velocity of APs is decreased. In 1977, Matsumoto and Tasaki (1977) found a mathematical equation to calculate the speed of conduction in unmyelinated nerve fibers, which can be used to estimate the speed also in myelinated fibers. With this equation, the apparent inconsistency can be resolved:

$$v_{\text{axon}} \approx \sqrt {\frac{d}{{8 \cdot {\rho}C^{2} \cdot R^{*} }}} ,$$

where the *v*_axon_ is the conduction velocity, *C* is the membrane capacity,* d* is the diameter of the nerve, *R* ^*^ is the resistance of the membrane, *ρ* is the axoplasmic resistance. According to the proposed model, the resting potential is increased due to a reduced open-state probability of the ion channels, therefore the resistance of the membrane (*R* ^*^) is increased. If membrane capacity (*C*), diameter of the axon (*d*) and axoplasmic resistance (*ρ*) are treated as constant in varying gravity, the increased resistance of the membrane leads to a decreased conduction velocity (v_axon_).

**Fig. 3:**
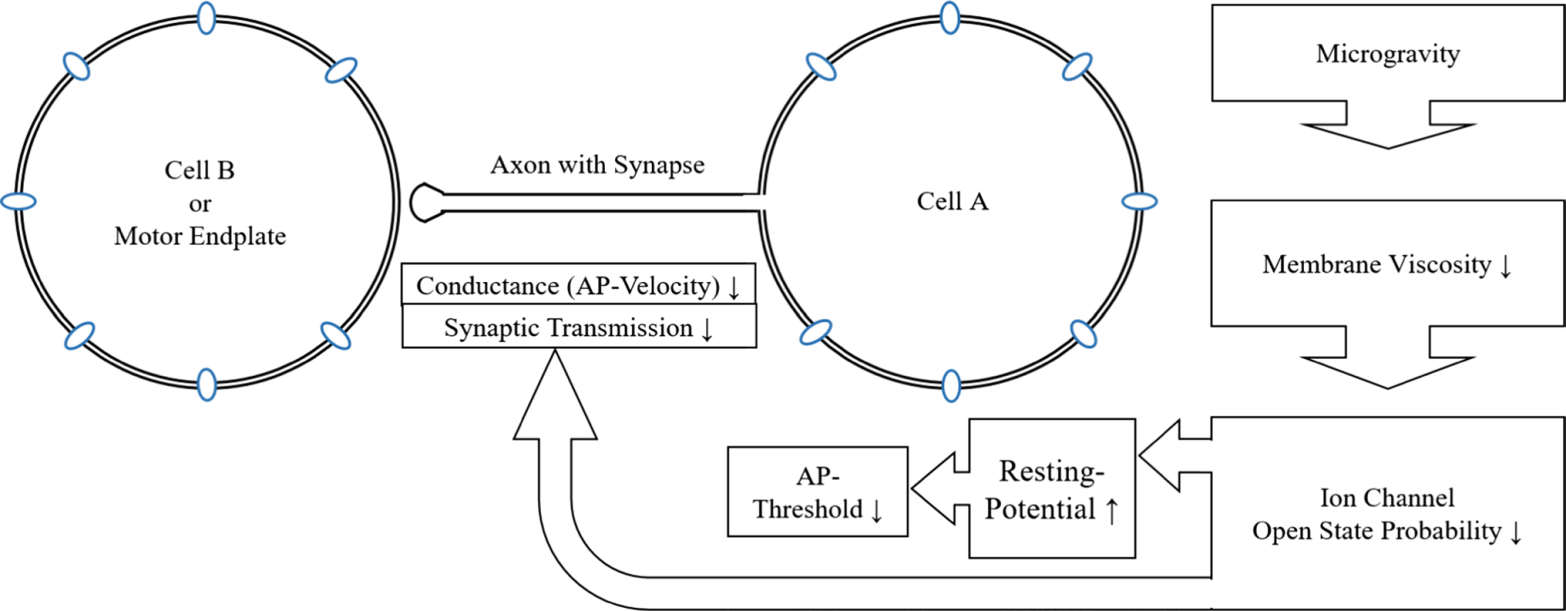
The final model from subcellular to multicellular level. Due to the changed membrane viscosity and the changed open-state probability, the cell depolarizes and the threshold to generate action potentials is reached more easily, but the AP velocity of the axons and the transmission speed at synapses in the motoric end plate are decreased, which seems to have a bigger impact than the reduced AP threshold

With this proposed model (Fig. 3), the short-term reaction of the sensorimotor system can be explained without any inconsistencies from the single neuronal cell up to the neuromuscular level. But of course, it opens up a lot of questions and open points, which will be discussed subsequently.

## Discussion

The aim of this review was to sum up and interconnect relevant publications about the adaptation of neuronal processes from the molecular to the (sub-) cellular level up to the complex neuromuscular system. Many separate in vitro and in vivo experiments on the different levels of the NS have been performed, each with a discrete result. Until now, no effort has been made to integrate these findings to either a working model, and/or to illustrate possible unresolved discrepancies, aiming for a better understanding of neuronal adaptation to variable gravity conditions and for a “roadmap” for future experiments. It is also an appeal for a more interdisciplinary approach to new experiments and to unite results of previously acquired data serving as a better comprehension of the gravity-induced challenges on organisms during prolonged manned space missions and to face them.

The presented short-term model interconnects results of separately working life science disciplines and these interconnections are based on assumptions, which have to be verified in future experiments. They are discussed as follows:

On the cellular level, it is not clear if membrane viscosity and the open-state probability of ion channels are the only gravity-sensitive parameters. For instance, the cytoskeleton of different cells is also affected by changes in gravity and therefore it is an additional sensor for g-load (Li et al. 2008), but the possible effect of a changed cytoskeleton on membrane fluidity has not been investigated in detail, yet. Until now, a model system (based on artificial membrane vesicles) and neuronal cells have been investigated separately and the authors showed a gravity-induced difference, which might be due to the absence of a cytoskeleton in the artificial vesicles (Sieber et al. 2014). It might also be possible that the lipid composition plays a role. The artificial vesicles were made of asolectin but the lipid composition of real cell membranes is more heterogeneous, depending on the cell type.

Two models for ion channels have been used to show the clear gravity dependence of the open-state probability of ion channels (Goldermann and Hanke 2001; Klinke et al. 2000), but there is no single channel data for real (neuronal) ion channels. Although the whole cell recordings from several research groups indicate that there is a gravity dependence of ion channels (Goldermann and Hanke 2001; Klinke et al. 2000; Schaffhauser et al. 2011; Richard et al. 2012), it is still unclear from the literature if all ion channel families, e.g., the relevant ion channel families for AP generation, react similar to changes in gravity. For the proposed model, this is assumed, but it still has to be investigated much more systematically. This has to be done with single-channel electrophysiology. Despite the challenge of doing this in microgravity, outside the ground-based laboratory, there are several promising approaches already indicating ion channel sensitivity to gravity (Wiedemann et al. 2011; Schaffhauser et al. 2011; Richard et al. 2012). In addition, the open-state probability is not the only relevant parameter. In regards to the completeness of nerve condition characteristics, e.g., the conductivity of the ion channels has to be investigated, as there are publications that indicate a dependence on gravity (Schaffhauser et al. 2011; Richard et al. 2012).

A detailed analysis of these parameters in the future would significantly help understanding the gravity dependence of cellular electrophysiology and ultimately the multicellular communication as in the neuromuscular system transferred to complex sensorimotor function. Based on the existing literature database, it is evident that molecular and cellular changes in response to gravity mentioned above affect the sensorimotor system in regard to human movement.

Immediate adaptations are reported in short-term experiments as well as in long-term investigations executed on the ISS or pre-post space flight, respectively. Analysis of motor and sensory responses regarding their timing and shaping (Ritzmann et al. 2015, 2016; Sato et al. 2001; Miyoshi et al. 2003; Nomura et al. 2001; Ohira et al. 2002; Kramer et al. 2013; Davey et al. 2004) demonstrate that NS function for muscle activation is changed and these changes most probably rely on molecular dysfunction: when axons conduct AP more slowly, consequently the motor response and muscle contraction are delayed (Ritzmann et al. 2016). Regarding human space flight, this is known to be a limitation for a safe return to earth as well as stopovers on other planets: for practical issues such as movement precision and control required for fall prevention or force generation, the cellular changes impact space mission safety (Blottner and Salanova 2015). Likewise, smaller neuromuscular responses as demonstrated by changes in reflex and motor response amplitude are associated with a reduction of muscle force (Aagaard 2003).

Based on gravity-induced changes in frequency originated on the subcellular level, this is also of considerable relevance: a reduced muscle response concomitant with a slowed down reaction negatively impacts motor control in daily relevant activities, such as in gait, posture control, or fine motor tasks (Layne et al. 2001; Bloomberg et al. 1999; Paloski et al. 1993; Mulavara et al. 2010).

This is exactly where security debates and countermeasure development move into focus: as reported in many space experiments, astronauts suffer from motor dysfunction associated with neuromuscular degradations and a performance decline after their return to earth (Blottner and Salanova 2015; Mulavara et al. 2010; Hargens et al. 2012; Clark and Bacal 2008).

Thereby, in cohorts of astronauts and cosmonauts with stays in space for 10–241 days, a sustaining increase of amplitudes (Reschke et al. 1986; Kozlovskaya et al. 1981; Grigoriev and Yegorov 1990; Baker et al. 1976), neuromuscular latency (Davey et al. 2004; Ruegg et al. 2003), and IPIs (Ruegg et al. 2003) concomitant with decreased PNS thresholds (Kozlovskaya et al. 1981; Grigoriev and Yegorov 1990) for H-reflexes, stretch and vibration reflexes after returning to earth could be demonstrated. Importantly, adaptations persisted beyond weightlessness for up to 2 weeks of earth life after space. As astronauts suffer from sensorimotor impairments associated with gravity-dependent changes in the nervous system, which limit the duration of space-stays (Edgerton et al. 2001), this concern is a major issue for the space agencies. Achievements of critical task under variable gravitation conditions depend on sensorimotor function (Edgerton et al. 2001). They are crucial for a safe space flight and return to earth.

However, there are limitations to the model that need to be considered: in vitro studies revealed contradictory results concerning the amplitude and latency of reflexes. Due to different methodologies, the outcomes are hardly comparable and a conclusive statement integrated into our working model is still speculative. Furthermore, the model does not take into account possible changes in nerve geometry, as there are electrophysiological properties as axoplasmic resistance and other electrical parameters. However, up to now, there are no experiments that have been performed focusing on this point, although it might be possible to investigate this with single cells, nerve fibers, or tissue samples of animals and humans.

Furthermore, the model only takes into account the changes of the electrophysiological component of neuromuscular latency. Of course, the sensorimotor reflex system has more components than that. Surely the electrochemical coupling in the neuronal synapses and the motoric end plate must be taken into account, as the findings on the IPIs indicate that this chemical component is also affected by gravity (Ritzmann et al. 2016). Experiments have to be designed that focus on receptor–ligand interactions under varying gravity conditions to clarify the possible gravity dependence of this process, as it might have a huge impact on neuronal and neuromuscular communication.

Besides neuroplasticity of the gravity-induced cell physiology, modulations in neural excitation could also be causal for the in vivo experimental outcomes. While cell physiology involves molecular adaptation based on electrochemical changes of the neural cell body or axon (Goldermann and Hanke 2001; Sieber et al. 2014; Kohn 2012), in contrast the excitability of reflexes and motor responses rely on a non-persisting phenomenon caused by spontaneous and task- or environment-specific inhibition or facilitation of neuronal pathways (Crone et al. 1990; Zehr 2002; Aagaard 2003).

Although an interlink of the observed gravity effects on the above-mentioned subcellular structures and the resulting nerve’s level of depolarization with the timing of reflexes and motor responses is apparent: the less fluid the membrane and the less open the ion channels are, the slower the action potentials will be transmitted via the axon. Hence, the period of the reflex latency, the duration, and the inter-peak-intervals as they occurred in reduced gravitation below normal gravity are longer. Consequently, the overall decrease in timing in micro-, lunar, and Martian gravity compared to earth and hypergravity most probably relies on gravity-induced cellular changes in neurons. Nevertheless, for gravity-dependent threshold changes and adaptations in amplitude, the underlying origins are less clear. Besides gravitational cell physiology as illustrated in the model, modulated excitations such as pre- and postsynaptic inhibition or facilitation should be taken into account (Ritzmann et al. 2015; Zehr 2002; Kohn 2012). Modulated proprioceptive sensory feedback and related central changes in motor commands descending from brain structures may have caused an inhibition of spinal reflexes in microgravity and a facilitation in hypergravity (Ritzmann et al. 2015; Davey et al. 2004). Vestibular, visual, and somatosensory input is altered in varying gravity (Layne et al. 2001; Homick and Reschke 1977; Paloski et al. 1993; Bloomberg et al. 1997) reflected by a highly reduced vestibule-somatosensory feedback concomitant with a predominance in vision for microgravity conditions (Layne et al. 2001; Paloski et al. 1993; Bloomberg et al. 1997). This may also have a large impact on motor commands and the inhibition or facilitation of Ia afferent pathways. For a more conclusive statement to clarify the origin of timing and shaping of reflex adaptations, further experiments are mandatory.

### Conclusions

The prospect of a sustainable overview and proper understanding of the gravity-dependency of the NS requires a number of new investigations. A lack of knowledge can be reduced by interdisciplinarity, including the gravitational influence on the cytoskeleton and conductivity of the ion channels of nervous cells as well as experiments in the motoric end plate. Moreover, novel approaches including the brain and peripheral circuitries using electrophysiology with an emphasis on long-term adaptations have the potential to further clarify if excitability changes are gravity-dependent and influence motor control.

In contrast to in vivo data, there is basically no data for cellular long-term adaptation processes as the technical and biological requirements for cellular long-term experiments in microgravity are challenging. Nevertheless, this topic should be addressed in the future to be able to extend the short-term adaptation model with the long-term adaptation mechanisms.
